# Clinical spectrum of non-alcoholic fatty liver disease in patients with diabetes mellitus

**DOI:** 10.1371/journal.pone.0236977

**Published:** 2020-08-21

**Authors:** Kaina Chen, Wei Kwan Sng, Joanne Hui-Min Quah, Jin Liu, Bee Yen Chong, Hwee Khim Lee, Xue Fei Wang, Ngiap Chuan Tan, Pik-Eu Chang, Hong Chang Tan, Yong Mong Bee, George Boon Bee Goh

**Affiliations:** 1 Department of Gastroenterology & Hepatology, Singapore General Hospital, Singapore, Singapore; 2 Duke NUS Medical School, Singapore, Singapore; 3 SingHealth Polyclinics, Singapore, Singapore; 4 Department of Endocrinology, Singapore General Hospital, Singapore, Singapore; Kaohsiung Medical University Chung Ho Memorial Hospital, TAIWAN

## Abstract

**Background:**

Non-alcoholic fatty liver disease (NAFLD) is increasingly widespread with an overall global estimated prevalence of 25%. Type 2 diabetes Mellitus (T2DM) is a key contributor to NAFLD progression and predicts moderate-severe liver fibrosis and mortality. However, there is currently no uniform consensus on routine NAFLD screening among T2DM patients, and the risk factors of NAFLD and advanced fibrosis among T2DM patients remain to be clarified fully.

**Aim:**

We explored the prevalence, clinical spectrum, and risk factors of NAFLD and liver fibrosis among T2DM patients.

**Methods:**

This is a cross-sectional study that enrolled subjects from a primary care clinic and a diabetes centre in Singapore. Subjects aged 21 to 70 years of all ethnic groups with an established T2DM diagnosis were included. Subjects with chronic liver diseases of other aetiologies were excluded. All subjects underwent transient elastography for hepatic steatosis and fibrosis assessment. Their demographics, anthropometric measurements and clinical parameters were collected. Statistical analysis was performed using STATA/SE16.0 software.

**Results:**

Among 449 enrolled T2DM subjects, 436 with complete data and valid transient elastography results were analysed. Overall, 78.72% (344/436) of the T2DM subjects had NAFLD, of which 13.08% (45/344) had increased liver stiffness. Higher ALT level (OR = 1.08; 95% CI: 1.03–1.14; p = 0.004), obesity (BMI ≥ 27.5 kg/m2, OR = 2.64; 95% CI: 1.28–5.44; p = 0.008) and metabolic syndrome (OR = 4.36; 95% CI 1.40–13.58; p = 0.011) were independent factors associated with increased CAP (NAFLD). Higher AST level (OR = 1.06; 95% CI: 1.02–1.11; p = 0.008), CAP value (OR = 1.02; 95% CI: 1.00–1.03; p = 0.003), lower platelet count (OR = 0.99; 95% CI: 0.98–1.00; p = 0.009) and concomitant hypertension (OR = 4.56; 95% CI: 1.18–17.62; p = 0.028) were independent factors associated with increased liver stiffness.

**Conclusions:**

Our study demonstrated a considerably high prevalence of NAFLD among T2DM patients, with the proportion of advanced liver fibrosis among T2DM NAFLD patients much higher than the general population. Given that NAFLD is largely asymptomatic, increased awareness and vigilance for identifying NAFLD and increased liver stiffness among T2DM patients should be advocated.

## Introduction

Non Alcoholic Fatty Liver Disease (NAFLD) is the most prominent cause of liver disease worldwide, with global estimated prevalence of 25% among adults [1]. More recently, the prevalence of NAFLD in Asia was reported to be 29.32%, with the pooled annual incidence at 50.9 cases per 1000 person-years [2]. It is associated with considerable clinical burden with the potential development of advanced fibrosis, liver cirrhosis, and hepatocellular carcinoma (HCC) [3, 4].

Type 2 Diabetes Mellitus (T2DM) is intricately intertwined with NAFLD progression and is commonly acknowledged as an independent predictor of moderate-severe liver fibrosis [5], in addition to overall and liver-related mortality [6–8]. In previous studies, up to 70% of T2DM patients were found to have NAFLD and of more concern, up to 20% reported with clinically significant fibrosis [5, 9]. In Singapore, with the prevalence of T2DM forecasted to increase exponentially from 7.3% in 2005 to 25% in 2050 [10], the burden of NAFLD is anticipated to be considerable in the coming decades.

NAFLD has long been regarded as a liver manifestation of metabolic syndrome. Despite the close association between NAFLD and T2DM, there is currently no uniform consensus on routine NAFLD screening among T2DM patients from major academic societies [11]. Nonetheless, there is increasing awareness of the high index of suspicion for NAFLD and NASH in patients with type 2 diabetes (T2DM). The gold standard for diagnosis and staging of NAFLD is liver biopsy, which is not well accepted by patients due to its invasive nature and consequently potential risks, and the accuracy is limited by sampling errors and interobserver variations [12, 13]. This has largely limited its use in investigating the true prevalence of NASH, the severe form of NAFLD and liver fibrosis in large cohorts. This has been, to a certain extent, rectified with the development of transient elastography (TE), which is now a well-established non-invasive modality to assess hepatic steatosis and fibrosis. It has high accuracy and has been utilized in assessing NAFLD fibrosis in community-based large populations [9, 14, 15].

In this study, we aim to investigate the prevalence and factors associate with hepatic steatosis and advanced liver fibrosis among subjects with T2DM.

## Materials and methods

### Study population and data collection

This was a prospective cross-sectional study enrolling subjects with T2DM consecutively from a primary care clinic (Outram Polyclinic) and the Endocrinology clinic from Diabetes & Metabolic Disease Centre (DMC) in Singapore General Hospital (SGH). These were clinics adept in the management of the whole spectrum of T2DM, incorporating multidisciplinary, comprehensive medical care and regular follow up for patients with T2DM. Enrolled subjects had not been previously been evaluated for NAFLD by gastroenterologists. The recruitment of subjects took place from 30 November 2015 to 19 October 2017.

Subjects aged 21 to 70 years of all ethnic origins with an established diagnosis of T2DM were included. T2DM was diagnosed in patients with typical symptoms and any one of the following present: 1) random plasma glucose ≥ 11.1mmol/L (200mg/dL); 2) fasting plasma glucose ≥ 7.0 mmol/L(126mg/dL); 3) 2-hour post-challenge plasma glucose ≥ 11.1mmol/L. In asymptomatic patients, diagnosis was confirmed if two different tests reach the diagnostic thresholds above. At the time of this study, HbA1C was not a recommended screening test for T2DM in Singapore because its correlation with average glucose was not consistent in different Asian ethnic groups [16]. Separately, metabolic syndrome was defined based on International Diabetes Federation (IDF) consensus (https://www.idf.org/e-library/consensus-statements/60-idfconsensus-worldwide-definitionof-the-metabolic-syndrome.html). Exclusion criteria included pregnant subjects, subjects with implantable electronic devices, known case of chronic liver disease of other etiologies, such as viral hepatitis B / C infections, autoimmune liver disease, drug related liver disease or significant alcohol consumption (>21 units / week for males, and >14 units / week for females). Subjects were initially screened by physicians or study team members for eligibility, and eligible patients were invited to SGH for a single study visit. At the study visit, detailed assessment using a case report form was performed for each subject. Information pertaining to demographic data, anthropometric measurements, comorbidities, basic blood tests as well as transient elastography examination were collated from all enrolled subjects. Body mass index (BMI) was calculated as body weight (kg) divided by body height (m^2^). Obesity was defined as BMI ≥ 27.5 kg/m^2^ as recommended by Singapore MOH obesity clinical practice guideline [17]. Fibroscan examination was performed at the same study visit. We used increased controlled attenuated parameter (CAP) value of more than 248 dB/m to define the presence of ≥S1 hepatic steatosis and Liver Stiffness Measurement (LSM) of 9.6 kPa or more for ≥ F3 liver fibrosis [18, 19]. Performance of Fibroscan was based on manufacturer’s recommendations. The Fibroscan result was considered reliable if 10 or more valid measurements were acquired. A success rate of more than 60% and the ratio of the interquartile range to the median of 10 measurements (IQR/M) being 0.3 or less defined successful readings [18]. All subjects provided written informed consent prior to study participation. This study followed the ethical guidelines and was approved by the Institutional Review Board.

### Statistical analysis

All subjects with complete data after applying inclusion and exclusion criteria were incorporated for analysis. Chi-square (Fisher’s Exact) test, and T-test /Man-Whitney U tests for comparison of dichotomous data and quantitative variables were utilized, respectively. For comparison involving more than two groups, one-way ANOVA or Kruskal Wallis tests were used. Univariate and multivariate logistic regression were performed to identify factors associated with outcome. All statistical analysis was performed using STATA/SE16.0 software.

## Results

### Demographics and clinical characteristics

**Fig 1:**
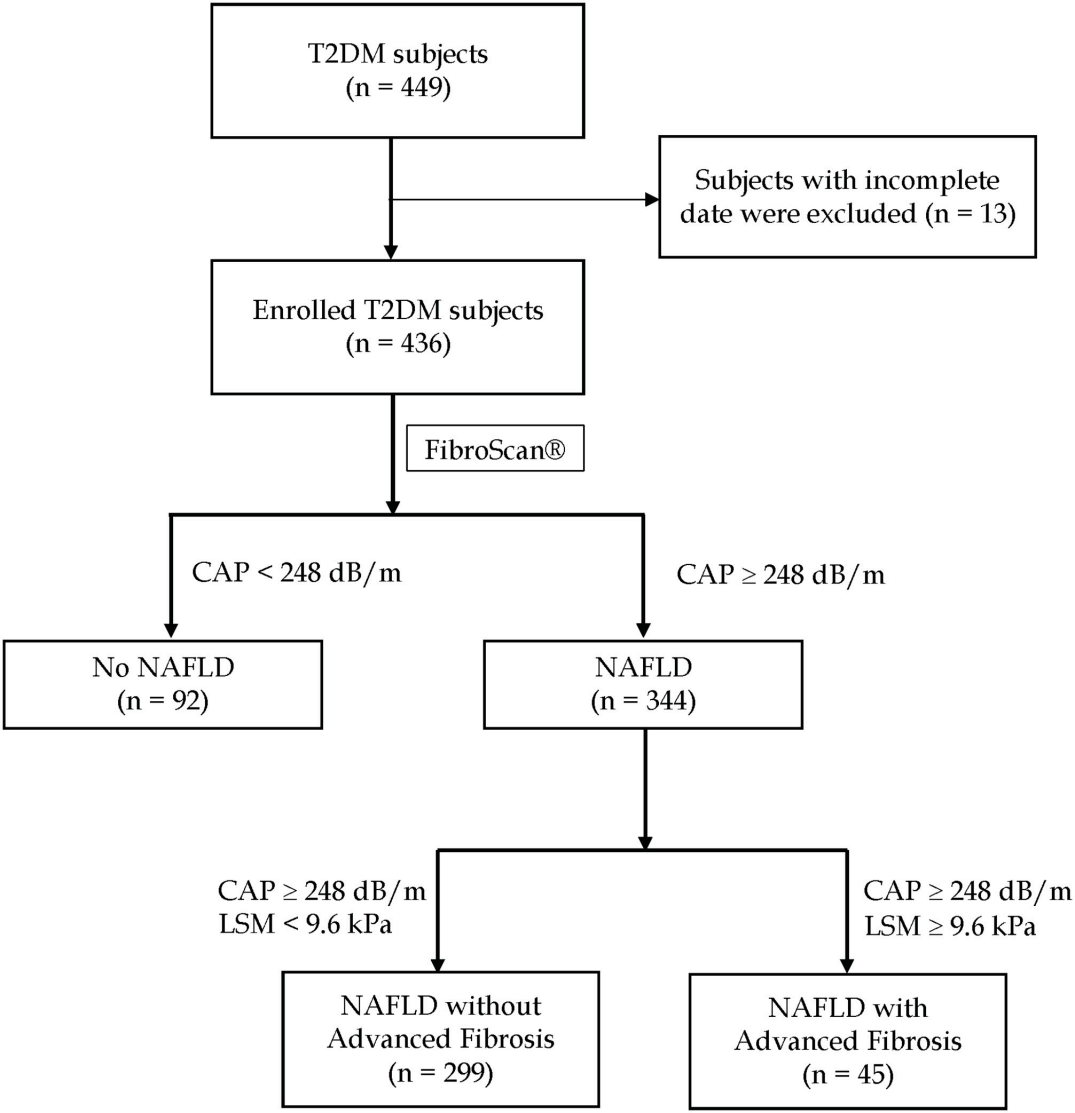
Prevalence of increased CAP (CAP > 248 dB/m) and increased liver stiffness (LSM ≥ 9.6kPa) in T2DM subjects.

A total of 449 subjects with T2DM were enrolled after inclusion and exclusion criteria were applied. Thirteen were excluded due to incomplete data. Overall, 78.72% (344/436) of the subjects had increased CAP suggestive of NAFLD (CAP > 248 dB/m), of which 13.08% (45/344) had increased liver stiffness (LSM ≥ 9.6kPa) (Fig 1). The mean age of our cohort was 58.52 (±9.49) years and 60.9% were males. The median duration of DM diagnosis was 10.40 (4.41–16.61) years and HbA1C was 7.2% (6.7%-8.1%). The average BMI of the cohort was 26.37 (±3.58) kg/m^2^, median CAP was 302.5 (258–346) dB/m and the LSM was 5.6 (4.6–7.5) kPa.

The demographics, biochemical results, comorbidities, and diet habits of T2DM subjects with (n = 344) and without (n = 92) increased CAP, as well as NAFLD with (n = 45) vs. without increased liver stiffness (n = 299) subjects were summarized in Tables 1 and 2.

**Table 1:** Demographics of T2DM subjects with or without increased CAP suggestive of NAFLD (CAP > 248 dB/m).

	NAFLD (N = 344)	Non-NAFLD (N = 92)	P value
Age (mean), years	58.48 (±9.50)	58.60 (±9.48)	0.915
gender, males	211 (61.34%)	54 (58.70%)	0.645
**BMI, kg/m**^**2**^	27.20 (±3.34)	23.27 (±2.62)	**<0.001**
**Waist circumference, cm**	88.12 (±9.85)	78.44 (±8.76)	**<0.001**
Smoker, yes	76 (22.09%)	24 (26.09%)	0.418
SBP, mmHg	132.01 (±17.21)	131.15 (±18.97)	0.677
**DBP, mmHg**	76.13 (±11.39)	73.40 (±10.03)	**0.037**
Fasting plasma glucose, mmol/L	8.19 (±2.73)	8.42 (±3.08)	0.544
HbA1C, %	7.62 (±1.51)	7.42 (±1.63)	0.265
**ALT,** IU/L	34.08 (±23.64)	22.01(±14.16)	**<0.001**
**AST,** IU/L	27.38 (±14.46)	22.27 (±9.76)	**0.002**
Albumin, g/L	43.84 (±2.66)	43.64 (±2.41)	0.517
Platelet, 10^9	261.65 (±66.83)	253.23(±64.97)	0.281
Total Cholesterol, mmol/L	4.94 (±8.28)	4.33 (±1.02)	0.508
Triglyceride, mmol/L	2.36 (±8.41)	1.31 (±0.80)	0.259
HDL, mmol/L	1.33 (±2.03)	1.35 (±0.97)	0.904
LDL, mmol/L	2.71 (±4.75)	2.45 (±5.10)	0.614
***Comorbidities***			
DM duration, years	11.46 (±8.53)	12.12 (±9.09)	0.522
**Hypertension, yes**	252 (73.26%)	55 (59.78%)	**0.012**
**Obesity (BMI≥27.5)**	162 (47.09%)	8 (8.7%)	**<0.001**
Hyperlipidemia, yes	281 (81.69%)	72 (78.26%)	0.457
**Metabolic syndrome, yes**	138 (54.55%)	8 (12.12%)	**<0.001**
Gout, yes	11 (3.2%)	3 (3.26%)	1.000
Stroke, yes	5 (1.45%)	3 (3.26%)	0.251
Ischemic heart disease, yes	48 (13.95%)	14 (15.22%)	0.758
***Diet Habits***			
**Soft drink / sweetened drink, at least 1 can / week**	120 (34.99%)	16 (17.39%)	**0.001**
Coffee, at least 1 cup / day	111 (32.27%)	35 (38.04%)	0.297
Fast food, at least 1 time / week	48 (13.95%)	9 (9.78%)	0.292

**Abbreviations** ALT: alanine aminotransferase; AST: aspartate transaminase; BMI: body mass index; DBP: diastolic blood pressure; DM: diabetes mellitus; HDL: high density lipoprotein; LDL: low density lipoprotein; SBP: systolic blood pressure

**Table 2:** Demographics of T2DM subjects with NAFLD, with or without increased liver stiffness (LSM ≥ 9.6kPa).

	LSM ≥ 9.6kPa (N = 45)	LSM < 9.6kPa (N = 299)	P value
Age (median), years	58.77 (±9.11)	58.43 (±9.57)	0.728
**gender, males**	35 (77.78%)	176 (58.86%)	**0.015**
**BMI, kg/m**^**2**^	28.69 (±2.68)	26.98 (±3.38)	**0.001**
**Waist circumference, cm**	93.5 (88–98)	88 (81.5–93.5)	**<0.001**
**Smoker, yes**	16 (35.56%)	60 (20.07%)	**0.019**
SBP, mmHg	136.67 (±20.11)	131.31 (±16.66)	0.052
DBP, mmHg	78.40 (±9.73)	75.79 (±11.59)	0.093
**Fasting plasma glucose, mmol/L**	**9.48 (±2.97)**	**7.98 (±2.64)**	**0.002**
HbA1C	7.77 (±1.44)	7.60 (±1.53)	0.492
**ALT, IU/L**	55.02 (±30.77)	30.78 (±20.50)	**<0.001**
**AST, IU/L**	39.00 (±12.77)	25.63 (±13.90)	**<0.001**
Albumin, g/L	43.64 (±2.85)	43.87 (±2.64)	0.597
**Platelet, 10**^**9**^**/L**	229.07 (±60.43)	266.55 (±66.46)	**<0.001**
Total Cholesterol, mmol/L	4.42 (±0.99)	5.02 (±8.90)	0.666
Triglyceride, mmol/L	1.98 (±1.37)	2.42 (±9.04)	0.756
HDL, mmol/L	1.17 (±0.28)	1.35 (±2.18)	0.593
LDL, mmol/L	2.38 (±0.93)	2.77 (±5.10)	0.633
**CAP, dB/m**	**349.44 (±36.97)**	**317.26 (±40.07)**	**<0.001**
***Comorbidities***			
DM duration, years	12.04 (±7.48)	11.38 (±8.68)	0.635
**Hypertension, yes**	39 (86.67%)	213 (71.24%)	**0.029**
**Obesity (BMI≥27.5)**	31 (68.89%)	131 (43.81%)	**0.002**
Hyperlipidemia, yes	38 (84.44%)	243 (81.27%)	0.45
Metabolic syndrome, yes	21 (65.63%)	117 (52.94%)	0.178
Stroke, yes	1 (2.22%)	4 (1.34%)	0.506
Ischemic heart disease, yes	48 (13.95%)	14 (15.22%)	0.758
***Diet Habits***			
Soft drink / sweetened drink, at least 1 packet/can per week	19 (42.22%)	101 (33.89%)	0.275
Coffee, at least 1 cup / day	37 (82.22%)	218 (72.91%)	0.184
Fast food, at least 1 time / week	6 (13.33%)	42 (12.05%)	0.898

**Fig 2:**
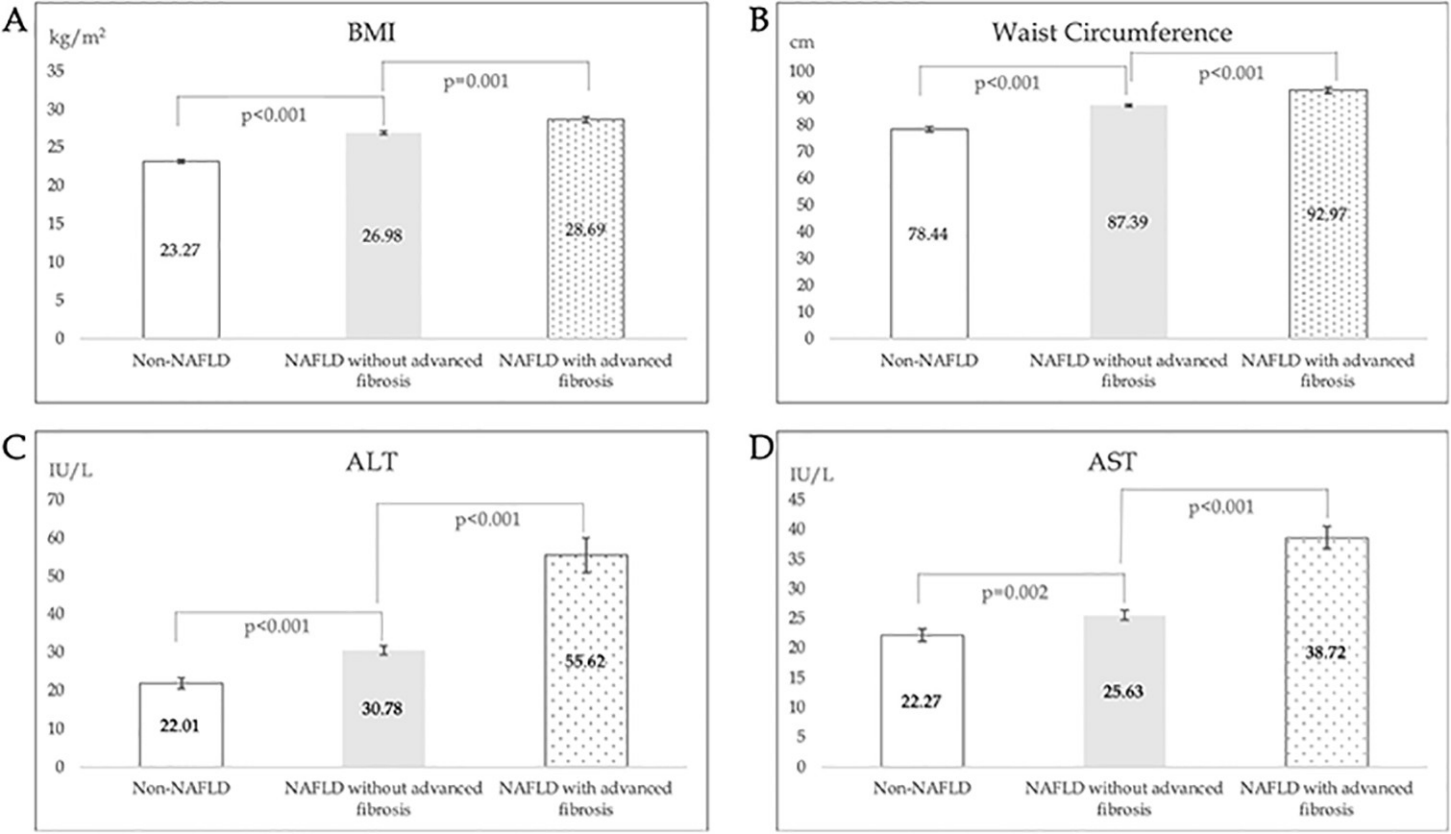
Comparison of BMI (A), waist circumference (B), ALT (C), AST (D) levels in Non-NAFLD, NAFLD with LSM < 9.6kPa and NAFLD with LSM ≥9.6kPa groups.

Relative to diabetic subjects without hepatic steatosis (non-NAFLD), BMI (27.2 vs 23.27 kg/m^2^, p<0.001), waist circumference (88.12 vs 78.44 cm, p<0.001), diastolic blood pressure (76.13 vs 73.40 mmHg, p = 0.037), ALT (34.08 vs 22.01 IU/L, p<0.001), and AST (27.38 vs 22.27 IU/L, p = 0.002) were significantly higher in NAFLD subjects (Fig 2). No significant difference in smoking status, lipid profile and platelet levels were observed. Considering comorbid conditions, higher proportions of NAFLD subjects were hypertensive (73.26% vs 59.78%, p = 0.012) obese (BMI≥27.5 kg/m^2^, 47.09% vs 8.7%, p<0.001) and had metabolic syndrome (54.55% vs 12.12%, p<0.001). Duration of T2DM and HbA1C were not significantly different in the two groups. Separately, NAFLD subjects were more likely to drink at least 1 packet of soft drink or sweetened drink per week (34.99% vs 17.39%, p = 0.001) but no difference was observed in fast food or coffee ingestion.

In terms of increased liver stiffness (LSM ≥ 9.6kPa), more males (77. 78% vs 58.86%, p = 0.015) and smokers (35.56% vs 20.07%, p = 0.019) were observed in the increased liver stiffness group. Higher BMI (28.69 vs 26.98 kg/m^2^, p = 0.001), waist circumference (92.97 vs 87.39 cm, p<0.001), fasting plasma glucose (9.48 vs 7.98 mmol/L, p = 0.002), ALT (55.02 vs 30.78 IU/L, p<0.001), AST (39 vs 25.63 IU/L, p<0.00 1), and lower platelet (220.07 vs 266.55 10^9^/L, p<0.001) were observed in subjects with increased liver stiffness (Fig 2). Hypertension (86.67% vs 81.27%, p = 0.029) and obesity (8.89% vs 43.81%, p = 0.002) were more likely in the increased liver stiffness group, but no differences in comorbidities such as stroke or ischemic heart disease were observed. No significant difference in diet habits were observed.

### Factors associated with hepatic steatosis

Risk factors associated NAFLD included higher BMI, waist circumference, diastolic blood pressure, ALT, AST and TG levels (Table 3). Hypertension, obesity, metabolic syndrome and regular soft drink ingestion were also associated with NAFLD from univariate logistic regression. Multivariate analysis revealed that only ALT level (OR = 1.084; 95% CI: 1.03–1.14; p = 0.004), obesity (OR = 2.64; 95% CI: 1.28–5.44; p = 0.008) and metabolic syndrome (OR 4.36; 95% CI 1.40–13.58; p = 0.011) were independent risk factors for hepatic steatosis.

**Table 3:** Risk factors for hepatic steatosis among T2DM subjects.

	Univariate Analysis			Multivariate Analysis		
Variables	OR	95% CI	P value	OR	95% CI	P value
Age	1.00	0.97–1.02	0.915			
Male gender	1.12	0.70–1.78	0.645			
**BMI**	**1.54**	**1.39–1.70**	**<0.001**			
**Waist circumference**	**1.11**	**1.08–1.14**	**<0.001**	1.00	0.95–1.05	0.924
Smoker	0.80	0.47–1.37	0.419			
SBP	1.00	0.99–1.02	0.676			
**DBP**	**1.03**	**1.00–1.05**	**0.035**	1.01	0.97–1.05	0.618
Fasting glucose	0.93	0.88–1.07	0.543			
HbA1C	1.10	0.93–1.29	0.266			
**ALT**	**1.05**	**1.03–1.08**	**<0.001**	**1.08**	**1.03–1.14**	**0.004**
**AST**	**1.05**	**1.02–1.08**	**<0.001**	0.97	0.91–1.04	0.464
Albumin	1.03	0.94–1.12	0.516			
Platelet	1.00	1.00–1.01	0.281			
Total Cholesterol	1.15	0.89–1.47	0.293			
HDL	0.99	0.87–1.13	0.904			
LDL	1.03	0.90–1.18	0.666			
**Triglyceride**	**2.16**	**1.44–3.25**	**<0.001**	1.20	0.76–1.88	0.441
***Comorbidities***						
DM duration	0.99	0.97–1.02	0.522			
**Hypertension**	**1.84**	**1.14–2.98**	**0.013**	1.23	0.56–2.71	0.610
**Obesity**	**9.35**	**4.39–19.89**	**<0.001**	**2.64**	**1.28–5.44**	**0.008**
Hyperlipidemia	1.24	0.70–2.18	0.458			
**Metabolic syndrome**	**8.70**	**3.99–18.97**	**<0.001**	**4.36**	**1.40–13.58**	**0.011**
Stroke	0.44	0.10–1.87	0.264			
Ischemic heart disease	0.90	0.47–1.72	0.758			
***Diet habits***						
**Soft drink 1 packet / week**	**2.56**	**1.43–4.58**	**0.002**	1.89	0.80–4.46	0.149
Coffee 1 cup/day	1.15	0.67–1.98	0.622			
Fast food 1 time / week	1.50	0.70–3.17	0.294			

### Factors associated with increased liver stiffness

For increased liver stiffness, univariate analysis identified a number of risk factors including male gender, smoker, higher BMI, waist circumference, fasting glucose, CAP value, ALT and AST levels, lower platelet level, comorbidity of hypertension, and obesity (Table 4). Only higher AST level (OR = 1.06; 95% CI: 1.02–1.11; p = 0.006), CAP (OR = 1.02; 95% CI: 1.00–1.03; p = 0.044), lower platelet level (OR = 0.99; 95% CI: 0.98–1.00; p = 0.017), and concomitant history of hypertension (OR = 4.63; 95% CI: 1.19–17.94; p = 0.027) were independent risk factors from multivariate analysis.

**Table 4:** Risk factors for increased liver stiffness (LSM ≥ 9.6kPa) among T2DM subjects with NAFLD.

	Univariate Analysis			Multivariate Analysis		
Variables	OR	95% CI	P value	OR	95% CI	P value
Age	1.00	0.97–1.04	0.822			
**Male gender**	**2.45**	**1.17–5.12**	**0.018**	1.69	0.55–5.20	0.356
**BMI**	**1.1**	**1.06–1.29**	**0.002**			
**Waist circumference**	**1.08**	**1.03–1.11**	**<0.001**	0.98	0.92–1.05	0.572
**Smoker**	**2.20**	**1.12–4.31**	**0.022**	1.62	0.60–4.34	0.340
SBP	1.02	1.00–1.04	0.053			
DBP	1.02	0.99–1.04	0.160			
**Fasting glucose**	**1.18**	**1.06–1.32**	**0.003**	1.14	1.00–1.30	0.051
HbA1C	1.07	0.88–1.31	0.491			
**ALT**	**1.04**	**1.02–1.05**	**<0.001**	1.00	0.97–1.02	0.867
**AST**	**1.05**	**1.03–1.08**	**<0.001**	**1.06**	**1.02–1.11**	**0.008**
Albumin	0.97	0.86–1.09	0.596			
**Platelet**	**0.99**	**0.98–1.00**	**<0.001**	**0.99**	**0.98–1.00**	**0.009**
Total Cholesterol	0.95	0.69–1.31	0.747			
HDL	0.56	0.17–1.81	0.332			
LDL	0.91	0.62–1.34	0.636			
Triglyceride	0.99	0.92–1.06	0.773			
**CAP**	**1.02**	**1.01–1.03**	**<0.001**	**1.02**	**1.01–1.03**	**0.003**
***Comorbidities***						
DM duration	1.01	0.97–1.05	0.634			
**Hypertension**	**2.62**	**1.07–6.42**	**0.035**	**4.56**	**1.18–17.62**	**0.028**
**Obesity**	**2.84**	**1.45–5.56**	**0.002**	2.89	0.96–8.62	0.058
Hyperlipidemia	1.25	0.53–2.95	0.608			
Metabolic syndrome	1.70	0.78–3.69	0.182			
Stroke	1.68	0.18–15.34	0.648			
Ischemic heart disease	0.94	0.38–2.36	0.898			
***Diet Habits***						
Soft drink 1 packet/wk	1.43	0.75–2.70	0.276			
Coffee 1 cup/day	1.72	0.77–3.85	0.188			
Fast food 1 time / wk	0.94	0.38–2.36	0.898			

## Discussion

The prevalence of T2DM in Singapore had doubled since 1980 and is a major public health concern that accompanies our aging society [20]. NAFLD is closely related to insulin resistance, and it’s increasingly recognized in Asia where the prevalence is approaching 30% and likely to continue to rise [2]. The clinical burden from NAFLD-related complications is expected to be considerable, because T2DM is known to be an accelerating factor for NAFLD progression and associated with increased mortality [5, 7, 21]. NAFLD is largely an asymptomatic condition and only manifests at late stage of liver disease. Indeed, previous studies had suggested that the general public may not be familiar with the risks associated with NAFLD, particularly in vulnerable subjects [22, 23]. In addition, the perceived lack of treatment for NAFLD by the public may add a further barrier to timely diagnosis and intervention. Consequently, improving public awareness of NAFLD and education of risk recognition and pre-emptive treatment in select populations such as T2DM patients may alleviate the onslaught of the NAFLD epidemic.

Our study demonstrated a remarkably high proportion of NAFLD among T2DM patients, which is echoed by other reported T2DM populations using TE [9, 24]. Our study did not find a correlation between NAFLD and HbA1C level or duration of DM. However, we acknowledge that a single HbA1C reading may not objectively assess overall T2DM control/severity, hence our inability to comment definitely about the relationship between NAFLD and DM severity. Factors independently associated with hepatic steatosis were higher ALT level, metabolic syndrome and obesity (BMI ≥ 27.5 kg/m^2^), which were consistent with earlier studies. Other reported factors from those studies such as higher triglyceride level and diastolic blood pressure were found to be significant from univariate, but not multivariate logistic regression in our study [9, 25]. Consumption of soft drink (sugar-sweetened beverage) at least one packet per week was a significant factor associated with NAFLD (OR = 2.56; 95% CI: 1.43–4.58; p = 0.002) among T2DM patients. Soft drink had been reported to be a risk factor for metabolic syndrome and increased the risk of mortality [26]. While the exact mechanism remains unclear, evidence suggest that it might be linked to increased insulin resistance and habitual preference of sweets. Indeed, a recent systematic review and meta-analysis demonstrated that sugar-sweetened beverage consumption significantly increased the risk of NAFLD in a dose-dependent fashion. At a quantity of at least 1 cup per week (moderate-high dose), the relative risk of NAFLD increased at least 26%, and up to 53% in patients who drank at least 1 cup per day [27]. This reiterates the importance of educating and fostering of healthy dietary patterns in the primary prevention of NAFLD.

The prevalence of increased liver stiffness suggestive of ≥ F3 disease was 13.08% among NAFLD and 10.32% among all T2DM subjects, which was much higher than that of the general population, echoing earlier studies [15, 25, 28, 29]. The substantial numbers of subjects with increased liver stiffness is concerning, especially since there are usually no associated symptoms. However, en masse screening of subjects with diabetes may also not be feasible or cost effective. Therefore, it is important to identify accompanying additional risk factors which assist in selection of a high-risk group for further investigation. Our study confirmed that higher AST and CAP values, lower platelet, and hypertension were independently factors for increased liver stiffness in our T2DM patients [9, 15, 24, 29]. Age had been reported in several studies as an association factor with the development of liver fibrosis [14, 30], however this was not demonstrated in our study. Further sensitivity analysis using categorical age groups at cut-off of 45 years (OR = 1.45; 95% CI: 0.42–4.97; p = 0.558) and 65 years (OR = 0.81; 95% CI 0.41–1.74; p = 0.642) did not significantly change our findings. One possible explanation could be that the presence of T2DM accelerated the progression of NAFLD which lead to a higher prevalence of liver fibrosis independent of age, as suggested in a separate study reporting similarly the higher probability of liver fibrosis in T2DM patients independent of age [15].

The limitations of our study include its cross-sectional nature and absence of histological data. Instead, we used transient elastography (TE, FibroScan), a simple and fast modality, to evaluate hepatic steatosis and fibrosis in this cross-sectional study. Admittedly, a single TE result in our cross-sectional study may not be a true reflection of patient’s fibrosis status as it can be confounded by several factors such as obesity, alcohol/food consumption, or operator-dependence. Indeed, a prospective study by Lee et al investigating serial TE examinations in T2DM patients demonstrated that more than half of the patients had LSM reduction in 3 years [31]. Nevertheless, TE has been endorsed as an alternative to liver biopsy by international guidelines in guiding clinical management of chronic liver disease, including NAFLD [11, 32]. The diagnostic performance for NAFLD fibrosis was excellent using a cut-off value of 9.6kPa, with a positive likelihood ratio (PLR) of 8.9 and the AUROC of 0.93 for ≥F3 fibrosis [18]. Therefore, TE may be a valid and accurate modality in assessing NAFLD fibrosis in a real-world community-based setting where liver biopsy is not practical for all subjects. Future studies for patients with repeat fibroscan and/or liver biopsy results, correlated to liver outcomes, will be useful to evaluate the utility of serial fibroscan for diabetic patients.

## Conclusions

NAFLD and liver fibrosis was highly prevalent in our T2DM patients in the community and diabetes clinic. In particular, patients with higher AST and CAP values, lower platelet count or comorbidity of hypertension have higher risk for increased liver stiffness and should be considered for further assessment. Our study findings support the role of screening for severe NAFLD in selective subjects with T2DM. Further research is warranted to optimize the necessary screening platforms and workflows.

## Supporting information

S1 Data (XLS)
